# Diagnostics to support elimination of lymphatic filariasis—Development of two target product profiles

**DOI:** 10.1371/journal.pntd.0009968

**Published:** 2021-11-15

**Authors:** Kimberly Y. Won, Katherine Gass, Marco Biamonte, Daniel Argaw Dagne, Camilla Ducker, Christopher Hanna, Achim Hoerauf, Patrick J. Lammie, Sammy M. Njenga, Rahmah Noordin, Kapa D. Ramaiah, Reda Ramzy, Ronaldo G. Carvalho Scholte, Anthony W. Solomon, Ashley A. Souza, Jordan Tappero, Emily Toubali, Gary J. Weil, Steven A. Williams, Jonathan D. King

**Affiliations:** 1 Division of Parasitic Diseases and Malaria, Centers for Disease Control and Prevention, Atlanta, Georgia, United States of America; 2 Neglected Tropical Diseases Support Center, Task Force for Global Health, Decatur, Georgia, United States of America; 3 Drugs & Diagnostics for Tropical Diseases, San Diego, California, United States of America; 4 Department of Control of Neglected Tropical Diseases, World Health Organization, Geneva, Switzerland; 5 Global Project Partners, Oakland, California, United States of America; 6 Institute of Medical Microbiology, Immunology and Parasitology, University of Bonn, Bonn, Germany; 7 German Centre for Infection Research (DZIF), partner site Bonn-Cologne, Bonn, Germany; 8 Eastern and Southern Africa Centre of International Parasite Control, Kenya Medical Research Institute, Nairobi, Kenya; 9 Institute for Research in Molecular Medicine, Universiti Sains Malaysia, Penang, Malaysia; 10 Consultant LF Epidemiologist, Pondicherry, India; 11 National Nutrition Institute, Cairo, Egypt; 12 Neglected, Tropical and Vector-Borne Diseases Unit, Pan American Health Organization, World Health Organization, Washington, D.C., United States of America; 13 Global Health, Bill and Melinda Gates Foundation, Seattle, Washington, United States of America; 14 Neglected Tropical Diseases Division, United States Agency for International Development, Washington, D.C., United States of America; 15 Infectious Diseases Division, Washington University School of Medicine, St. Louis, Missouri, United States of America; 16 Department of Biological Sciences, Smith College, Northampton, Massachusetts, United States of America; University of Glasgow, UNITED KINGDOM

## Abstract

As lymphatic filariasis (LF) programs move closer to established targets for validation elimination of LF as a public health problem, diagnostic tools capable of supporting the needs of the programs are critical for success. Known limitations of existing diagnostic tools make it challenging to have confidence that program endpoints have been achieved. In 2019, the World Health Organization (WHO) established a Diagnostic Technical Advisory Group (DTAG) for Neglected Tropical Diseases tasked with prioritizing diagnostic needs including defining use-cases and target product profiles (TPPs) for needed tools. Subsequently, disease-specific DTAG subgroups, including one focused on LF, were established to develop TPPs and use-case analyses to be used by product developers. Here, we describe the development of two priority TPPs for LF diagnostics needed for making decisions for stopping mass drug administration (MDA) of a triple drug regimen and surveillance. Utilizing the WHO core TPP development process as the framework, the LF subgroup convened to discuss and determine attributes required for each use case. TPPs considered the following parameters: product use, design, performance, product configuration and cost, and access and equity. Version 1.0 TPPs for two use cases were published by WHO on 12 March 2021 within the WHO Global Observatory on Health Research and Development. A common TPP characteristic that emerged in both use cases was the need to identify new biomarkers that would allow for greater precision in program delivery. As LF diagnostic tests are rarely used for individual clinical diagnosis, it became apparent that reliance on population-based surveys for decision making requires consideration of test performance in the context of such surveys. In low prevalence settings, the number of false positive test results may lead to unnecessary continuation or resumption of MDA, thus wasting valuable resources and time. Therefore, highly specific diagnostic tools are paramount when used to measure low thresholds. The TPP process brought to the forefront the importance of linking use case, program platform and diagnostic performance characteristics when defining required criteria for diagnostic tools.

## Author summary

High quality diagnostic tools are an essential component of lymphatic filariasis (LF) programs. Currently, diagnostic tools are used by national programs to establish baseline endemicity, monitor progress of program interventions, determine when interventions can be stopped, and surveillance. For years, LF programs have relied on diagnostic tools that have been central to informing key program decisions but were not necessarily designed to undertake the roles for which they are used. Known limitations of existing diagnostic tools make it challenging to have confidence that program endpoints have been achieved. Target product profiles (TPP) provide product requirements to guide developers and manufacturers in their efforts to design tools fit for purpose. However, development of diagnostic tools used in public health programs requires consideration of aspects beyond those considered when developing diagnostic tools used for clinical diagnosis. The TPP process for two LF use cases brought to the forefront the importance of linking use case, program platform and diagnostic performance characteristics when defining required criteria for diagnostic tools.

## Introduction

Lymphatic filariasis (LF) is a mosquito-transmitted neglected tropical disease (NTD) caused by infection with filarial parasites (*Wuchereria bancrofti*, *Brugia malayi*, or *Brugia timori*) [1]. Over time, infection can lead to damage of the lymphatic vessels, leading to hydrocele, lymphedema, and elephantiasis. People who live with chronic and disabling manifestations of LF may experience reduced economic productivity and social stigma [2]. The Global Programme to Eliminate Lymphatic Filariasis (GPELF) aims to eliminate LF as a public health problem with a two-armed approach: stopping the spread of infection through mass drug administration (MDA) of antifilarial medicines and alleviating suffering among patients through morbidity management and disability prevention. There is little debate on the considerable progress toward LF elimination. As of 2019, 22 of 72 endemic countries had reduced infection levels below target thresholds and no longer required MDA nationally. The World Health Organization (WHO) has acknowledged 17 of these countries as having met criteria for eliminating LF as a public health problem [3]. It is estimated approximately 97 million cases have been prevented or cured through the strategies recommended by WHO, due in part to delivery of more than 8.2 billion treatments to more than 923 million people [3,4]. In addition, more than USD $100 billion in economic losses have been averted [5]. Despite this progress, critical limitations such as diagnostic tool deficiencies, inability to scale up interventions in some areas and paucity of surveillance guidance need to be addressed to reach established program goals [6].

In 2017, WHO recommended the combination of ivermectin, diethylcarbamazine and albendazole (IDA), for MDA in countries and territories not co-endemic for onchocerciasis or loiasis [7]. A single dose of this triple drug combination is more effective at clearing and sustaining microfilariae (Mf) clearance than the standard two-drug combinations used since the start of GPELF [8,9]. Sustained clearance of Mf indicates a potential sterilizing effect on adult worms, an outcome not observed when using combinations of albendazole with either diethylcarbamazine or ivermectin. As such, it is possible fewer rounds of MDA using IDA will be needed to interrupt transmission compared to two-drug combinations which are typically provided annually for five years or longer.

Currently, program progress is monitored by testing residents of communities at an administrative level, often a district, under treatment for the presence of circulating filarial antigen (CFA) for *W*. *bancrofti* and antifilarial antibodies (BmR1) for *Brugia* spp. The Alere Filariasis Test Strip (FTS; Abbott, United States), which measures CFA, and the Brugia Rapid Test (BRT; Reszon Diagnostics, Malaysia), which measures antifilarial antibodies, are used in areas endemic for *W*. *bancrofti* and *Brugia* spp., respectively. Demonstration that the population prevalence of positive tests for these analytes is below a defined threshold in a transmission assessment survey (TAS) is an indication that LF is no longer a public health problem in the area assessed [10]. While this monitoring approach was effective for the two-drug strategy, which is typically conducted at least five years after initiating MDA, follow-up evidence from initial IDA safety and efficacy studies showed persistence of CFA for five years after a single round of IDA-MDA even with sustained clearance of Mf [9,11]. Thus, measuring CFA alone may not be an adequate way to monitor LF elimination programs using IDA. While testing for Mf is possible, it is not ideal in program settings because of limitations in technical capacity, low sensitivity after MDA and the nocturnal periodicity of the parasite in many endemic settings, making blood collection inconvenient. To continue using the existing monitoring and evaluation (M&E) framework without modification, new diagnostic tools are urgently needed to detect the presence of viable worms following introduction of IDA.

Along with better tools to conduct M&E, and as more national programs reach established benchmarks and stop MDA, the importance of monitoring for resurgence through surveillance activities also increases. WHO recommends repeating the TAS twice at 2- to 3-year intervals after MDA has stopped. Demonstration that the number of antigen-positive children in each survey remains below the target threshold provides evidence that transmission is not sustainable. While the TAS is useful for stop-MDA decisions, it is not statistically powered to measure reductions in prevalence or incidence over time or to be a sensitive measure of recrudescence in transmission potential. Limitations of available diagnostic tests compound the survey design limitation. CFA can take 12 months or more to appear after incident infection and may persist several months to years after adult worms can no longer reproduce or have died [9,12,13]. Similarly, the presence of antifilarial antibodies may be indicative of prior exposure to *Brugia* spp. and may not represent an active infection or viable parasites [14–16]. Existing diagnostic tools have been adequate for mapping disease distribution and monitoring progress of interventions, but new diagnostics targeting analytes that represent recent exposure or pre-patent infection are needed to inform LF post-MDA and post-elimination surveillance.

As LF programs move closer to elimination targets, diagnostic tools capable of supporting the needs of the program are critical for success. For both stopping IDA and surveillance, known limitations of existing diagnostic tools make it challenging to have confidence that program endpoints have been achieved. The need for high quality diagnostics is not unique to LF, and critical gaps exist for many NTDs. As suggested in a recent publication, four important considerations are necessary when developing new diagnostic tools for NTD programs–(i) recognition that specificity determines the utility of diagnostic tools in low prevalence settings; (ii) consideration of the M&E framework in which the diagnostic tools will be used; (iii) willingness to move away from a single-test paradigm; and (iv) adaptation of M&E decision rules to reflect test performance [17]. As control and elimination targets were considered in development of a new NTD road map for 2021–2030, common gaps across the diverse NTD portfolio were highlighted [6]. There was recognition that a concerted effort was needed to address diagnostic deficiencies, or the NTD community would risk falling short of the new targets. In 2019, in acknowledgment of the identified priority, WHO established a Diagnostic Technical Advisory Group (DTAG) tasked with reviewing and prioritizing diagnostic needs for NTD programs, defining use-cases and target product profiles (TPPs) for needed tools, working with national NTD programs and implementing partners to support test development and validation, and providing WHO with guidance and recommendation on adoption of new diagnostics [18]. Subsequently, disease-specific DTAG subgroups, including one focused on LF, were established to follow a standardized process to develop TPPs and use-case analyses to be used by product developers. Here, we describe the development of two priority diagnostic TPPs for LF.

## Methods

**Fig 1:**
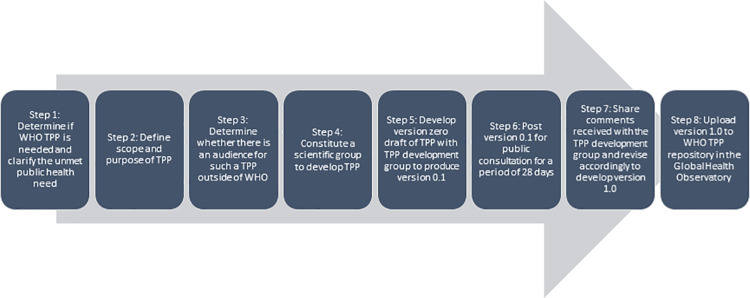
WHO Core TPP process.

Upon recommendation of the DTAG, WHO formed a group (known as the LF subgroup) of LF technical experts, end users and other stakeholders which met virtually in April 2020 to execute the process of developing TPPs for two priority use cases, stopping IDA and surveillance. TPPs were intended to facilitate expeditious development of new diagnostic assays addressing prioritized public health needs. Utilizing the WHO core TPP development process as the framework [18], and following well-established quality planning methodologies [19,20], the LF subgroup convened online a total of four times to discuss and determine attributes required for each use case (Fig 1).

TPPs for each use case considered the following parameters: product use, design, performance, product configuration and cost, and access and equity. Initial Draft 0 requirements in each TPP were selected based on landscape analyses, use case needs analysis and diagnostics performance modeling developed in late 2019 through a consultative process coordinated by WHO Department of the Control of Neglected Tropical Diseases and facilitated by a certified quality management expert. The LF subgroup critically reviewed and modified the zero draft where warranted. For certain elements in each use case, parameters were defined at the outset, and assumptions were made to move forward with sensitivity and specificity calculations (Tables 1 and 2). While there is no WHO target threshold for surveillance, researchers have proposed a provisional threshold of 5% antibody prevalence in children [21]. This provisional threshold was used for calculations in the surveillance TPP. The LF subgroup finalized the TPP details, and draft 0.1 TPPs were posted for 30 days on the WHO website for public comment in September 2020. A link to the public comment site was sent out to stakeholders including researchers, non-governmental organizations, ministries of health and other NTD program partners. Comments received were discussed at the final meeting of the LF subgroup in November 2020, and TPPs were revised accordingly to generate version 1.0 TPPs.

**Table 1:** Parameters and assumptions for use case 1: stopping IDA.

Calculations were based on testing a 1% infection prevalence threshold and considered two potential scenarios: • "Single test" approach in which a new assay would replace the current tools (i.e., FTS/BRT) in the TAS; and • "Decision confirmatory test" approach in which the new diagnostic test would be used as a decision confirmatory assay on individuals testing positive by FTS/BRT. Results of the confirmatory test would be used to make a program decision at a population level as opposed to an individual clinical treatment decision or confirmation of the first test result. Assumptions made for sensitivity and specificity calculations: • FTS/BRT is 90% sensitive for detecting Mf-positive individuals; FTS specificity 100% outside of *Loa loa* co-endemic areas • Survey design: 30-cluster; equal probability of selection • “Single test” approach: 80% power to correctly conclude that a defined population with a true prevalence ≤0.2% (ideal) or = 0% (minimum) is below the 1% threshold • “Decision confirmatory test” approach: 80% power to correctly conclude that a defined population with a true prevalence ≤0.5% (ideal) or ≤ 0.2% (minimum) is below the 1% threshold • α ≤5% (i.e., Type 1 error rate); meaning that using this diagnostic test, the survey would incorrectly conclude that prevalence in a defined population is below the 1% threshold <5% of the time

**Table 2:** Parameters and assumptions for use case 2: Surveillance.

Assumptions made in sensitivity and specificity calculations: • The diagnostic test would be used to identify evidence of transmission hotspots; calculations assume a lot quality assurance sampling approach where the goal is the upper 1-sided confidence interval of the prevalence is <5% • The calculations account for a finite population correction for village level prevalence; villages population sizes 300 to 5,000 people were considered • α ≤5% (i.e., Type 1 error rate); this means that using this diagnostic test, the survey would incorrectly conclude prevalence in a defined population is below the 5% threshold <5% of the time • 80% power to correctly conclude prevalence is below the 5% threshold in a defined population with a true prevalence ≤2% (ideal); and ≤1% (minimum)

## Results

Version 1.0 TPPs for two use cases, stopping IDA and surveillance, were published by WHO on 12 March 2021 within the WHO Global Observatory on Health Research and Development [22,23]. Select TPP features and their associated requirements are presented in Tables 3 and 4.

**Table 3:** Select characteristics of needed test to support stopping IDA decisions.

Feature	Ideal requirement	Minimum requirement
Target analyte	Antigen(s) or other biomarker(s) specific for *Wuchereria bancrofti*, *Brugia malayi* or *Brugia timori* and indicative of the capability to reproduce at the time of testing or thereafter. The absence of biomarker must indicate the worms are either not present, dead or permanently sterilized.	Same
Diagnostic/clinical sensitivity	"Single test" approach: > 60% "Decision confirmatory test" approach: > 85%	"Single test" approach: > 40% "Decision confirmatory test" approach: > 78%
Diagnostic/clinical specificity	"Single test" approach: > 99.7% "Decision confirmatory test" approach: > 96%	"Single test" approach: > 99.5% "Decision confirmatory test" approach: > 82%

**Table 4:** Select characteristics of needed test for surveillance of lymphatic filariasis.

Feature	Ideal requirement	Minimum requirement
Target analyte	Antibody(s) or other biomarker(s) specific for early exposure or pre-patent infection of *Wuchereria bancrofti*, *Brugia malayi*, or *Brugia timori*	Same
Diagnostic/clinical sensitivity	>99% sensitivity	>85% sensitivity
Diagnostic/clinical specificity	>99.8% specificity	>98.8% specificity

## Discussion

The GPELF strategy is predicated on the assumption that if antifilarial medicines are delivered consistently over a defined period, transmission will be reduced to a level at which it is no longer sustainable even in the absence of MDA. A clear threat to LF elimination is the inability to assess accurately if continued intervention is warranted. Prior to the launch and in the early years of GPELF, detection of Mf in peripheral blood was used routinely to identify individuals for treatment [24]. Detection of Mf in thick blood films served as an indication of viable adult worm infections and provided evidence needed to make treatment and programmatic decisions. However, logistical challenges were encountered because of the requirement for night blood collections. Furthermore, it was increasingly difficult to detect Mf in populations after multiple rounds of MDA [25]. Many of the limitations experienced with Mf detection were addressed with the introduction of tests to detect CFA. The detection of CFA served as a reasonable proxy for infection, and importantly, could be conducted with blood collected any time of the day, thus eliminating the need for night blood collections. The development of a rapid test to detect CFA in the 1990s was considered a turning point for LF elimination [26,27]. Logistical barriers of night blood collection for Mf detection were largely overcome, and the wide availability of a highly sensitive and specific CFA test gave confidence that program targets had been achieved. However, like the observed decline in Mf prevalence after treatment, antigenemia also declined in populations after multiple rounds of treatment [25]. Recently, the apparent lack of specificity of CFA tests in areas co-endemic for *Loa loa* and persistence of CFA in areas treated with IDA have posed problems for accuracy in decision making for surveillance and stopping MDA [9,13,28,29]. Despite known limitations, GPELF has relied on diagnostic tools that have been central to informing key program decisions but were not necessarily designed to undertake the roles for which they are used. However, limitations of the tools for use as multi-purpose diagnostics have largely been outweighed by their ability to drive program advances. Although the CFA test continues to be suitable in some settings such as areas that use one of the standard two-drug combinations, its use is inadequate in other settings. Hence, it is imperative to strive for efficient ways to strengthen programs, improve upon programmatic feasibility and develop tools fit for purpose. The need for high quality diagnostic tools to accurately assess the status of programs was recognized by the DTAG in 2019, highlighting the urgency to identify rapid solutions. The TPP development process allowed for careful consideration and a systematic approach to outline characteristics of diagnostic tools needed to overcome current barriers.

A common TPP characteristic that emerged in both use cases was the need to identify new biomarkers that would allow for greater precision in program delivery. While introduction of IDA represents potential to accelerate LF elimination, the full potential of the new drug regimen may not be reached if it is not coupled with improved ways to effectively determine when it is safe to stop MDA. In areas where multiple rounds of MDA have been conducted, antigen or antibody positive children identified in TAS likely represent recent exposure or infection. However, in areas where IDA is used, positive signals in TAS are challenging to interpret. The IDA TPP underscores the urgent need to identify alternative indicators to CFA and antifilarial antibodies to BmR1. Given the current M&E framework in which the diagnostic tools are used, accuracy and efficiency in decision making will be strengthened if infection with worms capable of reproduction can be differentiated from infection with sterile or dead worms that do not pose a threat to sustained transmission. Similarly, the surveillance TPP reflects a need to identify markers capable of accurately distinguishing signals that denote immediate threats from those representing historic or waning signals in the population. In the surveillance context, it is assumed that detecting the earliest opportunity to address recrudescence or ongoing transmission will be critical. Current evidence suggests antibody responses often appear months to years before CFA or Mf and could be an early warning signal during surveillance [12]. Importantly, antibody responses decline over time, and the absence of antibody responses provides evidence of the absence of transmission [30]. However, in the context of more recent transmission, it has been challenging to distinguish recent exposures from persistent responses to past exposures or infections. Additionally, existing antibody tests often lack the level of specificity needed [14,31,32], and some degree of non-specific reactivity is expected with most tests. This issue complicates the ability to determine an antibody threshold indicative of recent or ongoing transmission making interpretation of antibody results complex. For example, apparent antibody signals in LF non-endemic areas of Togo were higher than in LF endemic areas, making it difficult to determine if program action was required [33]. The LF DTAG subgroup recognized that discovery and validation of alternative markers may require significant time and effort. However, the high-risk nature of the research effort was viewed as essential to make necessary advances.

The utility of a diagnostic tool is often defined by its performance characteristics (e.g. sensitivity and specificity). While some of these parameters are mutable, high sensitivity and specificity are often considered optimal benchmarks to achieve. Defining the parameters of the IDA and surveillance TPPs required a shift from traditional considerations of ideal performance. Unlike some disease control and elimination programs (e.g. HIV), LF diagnostic tests are rarely used to make individual clinical diagnoses. Instead, tests used to make program decisions at a population level are applied according to a defined M&E framework. A typical TAS is conducted via cluster-based sampling of approximately 1,700 young children in a defined evaluation unit (EU) (e.g. district) [10]. Program decisions are made by comparing the observed number of positive test results in a survey against a target threshold. The observed number of positive results is dependent on the prevalence of positive tests, which includes true positives and those testing positive falsely. As such, additional considerations are necessary when interpreting test results since imperfections in performance of diagnostic tools can be compounded when applied across a surveyed population. When comparing survey results to a low threshold and using a diagnostic tool with <100% specificity, false positive results may drive an incorrect program decision, particularly in low prevalence settings, even if program interventions have been successful. In TAS, the number of false positives may lead to unnecessary continuation or resumption of MDA, thus wasting valuable resources and time. Therefore, highly specific diagnostic tools are paramount when used to measure low thresholds [17]. The sensitivity requirements defined in the IDA TPP appear low by conventional standards, but as LF infections become increasingly rare the sensitivity of a diagnostic test has very minimal impact on positive predictive value [17]. Allowing lower sensitivity does not automatically imply that the ability to make correct decisions based on survey outcomes will be poor. However, it is possible that survey designs may need to be adjusted either by increasing the sample size or by decreasing the critical cut off to ensure that the statistical parameters are within the desired range (e.g. 5% alpha error and 75% power in the TAS).

As performance requirements were determined for each use case, it became apparent that reliance on population-based surveys for decision making requires consideration of test performance in the context of such surveys. The TPP process brought to the forefront the importance of linking use case, program platform and diagnostic performance characteristics when defining required criteria for diagnostic tools. For the surveillance TPP, the absence of well-defined M&E guidelines led to the need to make several assumptions that may change as surveillance guidance is updated. For the surveillance TPP, an assumption was made that a standalone population-based survey will be conducted, and concurrently, a critical assumption was made that the EU definition is likely to change. With a shifting trend toward identification and response to hotspots to verify elimination of transmission, the geographic area to consider will shrink, resulting in assessment of relatively small communities as the unit of programmatic action (e.g. village). With this shift, survey sample sizes will be constrained, and performance requirements of diagnostic tools will be affected. Although the threshold assumed for the surveillance TPP (5%) was higher than the IDA TPP (1%), the defined sensitivity and specificity requirements were more stringent. The requirement to distinguish a signal that triggers a public health response from background reactivity (1–2%) led to the need for a diagnostic tool sensitive enough to detect true infections but also specific enough to exclude false positive test results. The parameters outlined in the surveillance TPP illustrate the need for test developers to understand test performance constraints that are critical to making effective decisions at the community level.

The need for highly specific diagnostic tools extends beyond LF and is applicable to other NTDs with low target thresholds, especially for disease programs that rely on population-based surveys when making decisions. Following the same standardized WHO process for developing TPPs, similar requirements for high specificity are reflected in recently published TPPs for onchocerciasis, schistosomiasis, and soil-transmitted helminths [34–36]. While there is inherent risk in setting high specificity standards, it is an opportunity to identify innovative and flexible solutions to meet criteria. It will be difficult to achieve such extreme standards in a single test, and the NTD community will have to evaluate alternative strategies such as testing approaches based on two tests. For the IDA TPP, a confirmatory test approach was considered. While a confirmatory test in this case was to confirm a programmatic decision and not an individual-level diagnosis, it still represents a multiple test strategy that will allow for specificity requirements of any single test to be relaxed. As new tools are developed, it is critical to standardize a framework in which new tools are validated. To ensure timeliness of results and comparability across settings, WHO can play an important role in guiding the standards by which tools are evaluated. The TPP development framework, guided by the DTAG and anchored by WHO, helps to assure a unified approach to identify and prioritize diagnostic needs according to program needs. Additionally, regular review of established TPPs will ensure that alignment with program needs is maintained.

The LF TPP development process confirmed the importance of prioritizing specificity requirements and the need to consider survey designs. While it was beyond the scope and mandate of the LF DTAG subgroup to consider changes to M&E frameworks, it emphasized the importance of maintaining close linkages between the DTAG and WHO M&E working group to ensure efforts are aligned for the benefit of NTD control and elimination programs. With clear M&E guidelines and appropriate diagnostic tools, national programs will be better able to plan activities to measure progress toward the 2030 goals.
